# Development of a High-Fidelity Framework to Describe the Process-Dependent Viscoelasticity of a Fast-Curing Epoxy Matrix Resin including Testing, Modelling, Calibration and Validation

**DOI:** 10.3390/polym14173647

**Published:** 2022-09-02

**Authors:** Johannes Gerritzen, Michael Müller-Pabel, Jonas Müller, Benjamin Gröger, Niklas Lorenz, Christian Hopmann, Maik Gude

**Affiliations:** 1 Institute of Lightweight Engineering and Polymer Technology, Technische Universität Dresden, Holbeinstr. 3, 01307 Dresden, Germany; 2 Institute for Plastics Processing, RWTH Aachen, Seffenter Weg 201, 52074 Aachen, Germany

**Keywords:** material model, viscoelasticity, cure, testing

## Abstract

Fast-curing epoxy resins enable substantial reduction of cycle times during production of thermoset polymer matrix composites. Due to the snap-cure behaviour, both characterisation and processing of these resins are associated with high complexity which motivates the development of a high-fidelity framework for the prediction of the process-dependent behaviour ranging from experiment to model validation. In order to determine influence of time, temperature, and degree of cure, a multitude of rheometer and dynamic mechanical analysis experiments are conducted and evaluated. Building on the experimental results, a material model based on a generalised Maxwell model is developed. It is calibrated on the results obtained in the tests and shown to describe the material’s behaviour with high accuracy under all investigated conditions. The model’s predictive capabilities are further tested by applying it to a dynamic mechanical analysis, exposing the model to previously unknown loading and temperature conditions. It is demonstrated that the model is capable of predicting such changing boundary conditions with high accuracy.

## 1. Introduction

Over the last decade, the development of fast-curing epoxy resins (EPs) and their introduction into industrial applications have paved the way for pioneering innovations in the field of composite-based lightweight structures [1]. This relatively novel class of reactive polymers enabled a significant reduction of cycle times and by this a substantially improved competitiveness of thermoset polymer matrix composites (PMC). Further reduction of costs and increase in robustness of the associated manufacturing processes inevitably require a deeper insight into the complex process-dependent material behaviour, which still poses a major challenge [1,2].

In this context, the development of cure-induced residual stresses and resulting dimensional changes received most scientific attention [2,3,4]. The importance of manufacturing process parameters for part quality was shown for a range of further composite key properties: based on cure experiments with neat resin in combination with online strain monitoring, Gross et al. derived modified cure schedules that enable a significant reduction of hydrostatic stresses during composite manufacturing [5]. The influence of residual stresses on matrix fatigue cracking of fast-curing EP-based composites was demonstrated by Joosten et al. [6]. Hunt et al. highlighted the importance of cure schedules for mode I fracture behaviour of toughened prepreg laminates [7]. The importance of different curing stages for the microscale adhesion between fibre and matrix was shown by ElKhoury and Berg [8]. Prussak et al. demonstrated the influence of different cure cycles on the resulting part distortion of hybrid PMC-steel laminates [9]. Although the described phenomena are not completely understood, there is some evidence that process-induced stresses play a key role for their explanation.

Residual stresses on the micro- and mesoscale are caused by the chemical resin shrinkage and the thermal expansion mismatch between resin and fibre as well as the pronounced anisotropy of PMC. Especially for the development of high-performance structures with demanding tolerance requirements, this multi-physical problem is usually tackled by process simulations [2,4,10,11]. This approach necessitates a detailed material model and corresponding input data which should capture the dependence on the relevant process parameters. In a recent publication, the current knowledge on determination of cure-dependent viscoelastic properties destined for material modelling has been reviewed [12]. In another recent publication, we suggested a methodology to measure and model the influence of cure, temperature, and pressure on the resin reaction kinetics, shrinkage, and thermal expansion of a fast-curing EP, which is applied for series production of large automotive structures using liquid composite moulding processes [13].

In this work, the focus will be on both the experimental determination and modelling of the cure- and time-dependent properties of the same resin system. Although a large body of literature was dedicated to this field of research, the debate about suitable model complexity is still ongoing [10,11]. Four main approaches for constitutive models can be identified, namely: (i) elastic, (ii) path-dependent, (iii) pseudo-viscoelastic, and (iv) viscoelastic models [10]. The choice of a constitutive model determines both the required experimental effort and the model applicability. Whilst elastic, path-dependent, and pseudo-viscoelastic approaches require very few input data, the development of truly viscoelastic models necessitates a more comprehensive testing program taking into account time, temperature, and degree of cure (DOC) [2,10,11]. Simplified models such as the path-dependent approach have the advantage of being computationally more efficient, but suffer from less flexibility [10,11,14] Viscoelastic models, on the other hand, may significantly improve the prediction of process-induced residual stresses as it was shown, e.g., by Brauner et al. [15]. Especially if non-conventional cure cycles are considered, their use is highly recommended [10]. For describing the viscoelastic relaxation behaviour during cure, two main approaches can be identified in the literature: the Kohlrausch–Williams–Watts-function [15,16] and the Prony-series approach [17,18,19,20].

Some of the previously published works on cure-dependent viscoelasticity of EP are based on experimental data that were determined by a single specimen geometry and a single test device [16,17,18,20]. This should be done with special care as the stiffness of EPs may span more than 12 decades during cure, while the admissible range of the different experimental setups is limited to a much narrower range [21]. Within the available works on cure-dependent viscoelasticity, parallel-plate rheology [17,18,22] and dynamic mechanical analysis (DMA) in bending mode [22,23] are most widely used as characterization methods, but also relaxation tests in bending mode were reported [16,20]. Typical rheometer devices are limited to a stiffness level in the range of 4 MPa when operated in oscillation mode [21,24], which means that the entire glassy regime of EP is out of their admissible range. In contrast, specimens for DMA tests must be initially solid, which excludes the measurement of the pre-gelation regime of reactive resins.

Given the importance of reliable experimental data for establishing a high-fidelity material model, this work is based on a four-stage approach being composed of (i) testing, (ii) modelling, (iii) calibration, and (iv) validation. The mechanical test conditions are selected with regard to the variable DOC and the variation of viscoelastic properties during processing. Furthermore, the structure–property-relationships of a fast curing EP are discussed in order to explain the experimental results and select a suitable model approach. The suggested viscoelastic model enables the realistic reproduction of the observed material behaviour taking into account all relevant dependencies.

## 2. Materials and Methods

The comprehensive experimental determination and modelling of cure-dependent viscoelastic properties is a complex [10,12] and rarely validated task [25]. The experimental program suggested here serves three purposes: input data for model derivation, calibration, and validation. By taking into account the available knowledge on ’avoiding bad data’ [21] and performing a careful theoretical interpretation, we strive for high-fidelity experimental input data. These data are used as basis for the derivation of a material model that takes the key dependencies into account. The model is subsequently calibrated on a subset of the experimental results and it is verified that input data can be reproduced well. The model’s predictive capabilities are validated by analysing an individual set of experimental results.

### 2.1. Materials

The material analysed in this study is EPIKOTE Resin TRAC 06150 (Hexion Inc., Columbus, OH, USA), a fast-curing EP used for series production of structural automotive composite components [26]. It consists of a bisphenol-A-based resin and an amine-based curing agent in a mass ratio of 100:24. According to the material safety datasheet, the hardener contains isophorone diamine, triethylenetetramine, and tris(dimethylaminomethyl)phenol. The maximum glass transition temperature ($T_{g}$) is 123 °C. To account for the different stiffness levels of the resin system during cure, two different specimen preparation methods and measurement geometries were used. For rheological measurements in parallel-plate geometry, the components were manually mixed at room temperature, filled into syringes and than directly injected between the pre-heated plates of the rheometer. The specimens used for relaxation measurements were prepared with the resin transfer moulding (RTM) equipment described in [13]. In this case, a modified cure schedule was chosen in order to yield solid samples with a DOC slightly above the point of gelation $\xi_{gel}$.

### 2.2. Experimental Investigations

In order to determine the process-relevant cure-dependent resin properties before and after gelation as well as below and above the $T_{g}$, the use of different specimen dimensions and loading modes is required [22]. As the rubbery modulus $G^{\infty }$ can be assumed to be time-independent [22], it is only analysed in dependence on DOC and temperature using isothermal oscillation tests in parallel-plate rheology. In contrast, the moduli in the glassy regime as well as during the relaxation are considered viscoelastic. Therefore, these will be determined in dependence on DOC, temperature and time by performing isothermal relaxation experiments in torsion mode on partially cured solid rectangular rods. In addition, a third type of experiment is performed to provide additional data for model validation. For this purpose, solid rectangular rods were manufactured and tested in a heated torsion DMA in order to demonstrate the model’s capability of predicting the viscoelastic properties under the combination of a different load type and continuously changing temperature. All measurements were preformed with an MCR 502 (Anton Paar Germany GmbH, Ostfildern, Germany).

#### 2.2.1. Isothermal Oscillation Experiments in Parallel-Plate Geometry

The pre-gelation regime of EP is characterised by initial low viscosity and rapidly increasing stiffness, as soon as the point of gelation is approached. To provide a suitable measurement window, parallel-plate rheology with a plate diameter of 25 mm and a gap of 1.5 mm is chosen. The tests are performed in oscillation mode with a frequency of 1 Hz and a shear amplitude of 0.1%. Assuming a device compliance of 0.008 radN m and an overall moment of inertia of 0.1 mN s${}^{2}$, this configuration is expected to deliver reliable results within a stiffness range of 200 Pa to 4 MPa [12,21]. The selected measurement temperatures of 80 and 100 °C correspond to realistic injection conditions during processing [13,26,27]. The point of gelation was determined by measurements in multiwave mode, in order to apply the Winter–Chambon criterion [12,28]. Frequencies of Hz = 1’ were applied. All stiffness data were transformed from time to DOC dependence by applying the reaction kinetic model.

#### 2.2.2. Isothermal Relaxation Experiments in Torsion Mode

In order to determine the viscoelastic properties in the post-gelation regime, neat resin plate material with a thickness of 2 mm was cured for 60 min at 60 °C in the RTM mould described in [13]. Rectangular rods with a cross-section of $2\;\times \;4\;{\mathrm{mm}}^{2}$ and a length of 40 mm were cut out of the plate. Assuming a device compliance of 0.008 radN m and a free specimen length of 30 mm, the configuration is expected to deliver reliable results up to a stiffness of 26 GPa [12]. After cutting, the specimens were post-cured in a calibrated oven to different DOC-levels. In order to avoid uncertainties related to the cure history, small pieces were cut from the partially cured specimen scans and used for differential scanning calorimetry (DSC) which yielded the results given in Table 1.

The relaxation experiments were performed isothermally with temperature intervals of 5 K, ranging from room temperature to 10 K below the current $T_{g}$ which was determined by DSC scans prior to the tests (see Table 1). A shear strain of 0.1% was applied within 0.1 s and kept constant for 5 min while the time-dependent stress response was recorded. The solid rectangular fixture (SRF) was used for clamping.

#### 2.2.3. Continuously Heated Oscillation Experiments in Torsion Mode

In order to evaluate the quality of the model prediction, a third type of experiment was conducted with the specimen geometry described in Section 2.2.2. In this case, a heated oscillation experiment with a shear amplitude of 0.1%, a frequency of 1 Hz, and a heating rate of 2 Kmin from room temperature to 150 °C was performed. The specimen was cured to full extent prior to the experiment. The described conditions are closely related to the cool-down of the composite material after the cure cycle is completed. This experiment gives an independent dataset, which can be employed to validate the overall model capability. Furthermore, the selected workflow represents an extra increase in complexity as the data used for model calibration were determined at constant temperatures.

### 2.3. Derivation of Governing Equations

In order to model the observed behaviour, firstly, the curing behaviour is modelled in Section 2.3.1. Secondly, the behavioural changes with the temperature are analysed in Section 2.3.2. Thirdly, the mechanical behaviour is investigated. For this, a distinction between two fundamentally different types is made:

- The underlying behaviour of the material in its equilibrium state. This behaviour is omnipresent and independent of loading or holding time and is discussed in more detail in Section 2.3.3.
- The strongly time- and rate-dependent behaviour observable in the conducted relaxation experiments. Since resulting stresses from this part of the material’s behaviour are overlaid with the equilibrium behaviour, they are often referred to as overstresses. This behaviour will be the focus of Section 2.3.4.

#### 2.3.1. Reaction Kinetics

The curing behaviour of the EP has been investigated in depth by the authors in [13]. The current reaction rate dξdt was accurately modelled with a parallel reaction of *n*th-order autocatalytic reaction coupled with diffusion-controlled curing based on the approach presented in [29], leading to the following formulation:

(1) dξdt=k1ξm(1−ξ)n1+k2(1−ξ)n2,with1ki=1ki,chem+1ki,diff,ki,chem=Aiexp(−EiRT)andki,diff=ki,diff*expC1,diff(T−Tg)C2,diff+T−Tg,

with the material and model parameters $m,n_{i},A_{i},E_{i},k_{i,diff}^{*},C_{i,diff}$; i=1,2 and universal gas constant *R*. The model parameters are taken from [13] and stated in Table 2.

#### 2.3.2. Time–Temperature Analogy

For modelling purposes it is assumed that the EP behaves thermorheologically simple as explained, e.g., in [30]. Therefore, the dependency of viscoelastic processes on the temperature is incorporated into an equivalent time $t^{*}$ defined per

(2) $$t^{*}=t\;\cdot \;a_{T}\left(T,\xi \right),$$

with the time–temperature shift factor $a_{T}$ depending on current temperature and DOC. The dependence on the temperature is modelled using a cubic formulation:

(3) $$\log\left|a_{T}\right|=\sum_{n=0}^{3}C_{n}\left(\xi \right)\;{\left(T-T_{g}\left(\xi \right)\right)}^{n},$$

with the DOC-dependent coefficients $C_{n}$. For states of curing not experimentally investigated, the values $C_{n}$ are obtained by interpolating between the two closest DOC-levels.

#### 2.3.3. Equilibrium Behaviour—Time-Independent

The investigated EP shows constant behaviour at extremely long equivalent loading times. Due to the network structure of the polymer, terminal flow is avoided and it can be assumed that after completion of the glass transition, no further relaxation phenomena will occur. Therefore, this behaviour is modelled by using a pure spring as rheological model, with the stiffness $G^{\infty }$.

The change in stress $\dot{\tau }$ resulting from a change in deformation $\dot{\gamma }$ can be calculated by

(4) $$\dot{\tau }=G^{\infty }\dot{\gamma }.$$

Since $G^{\infty }$ is defined to be independent of time, and time is considered to be interchangeable with temperature (cf. Section 2.3.2), $G^{\infty }$ has to be modelled as independent of both time and temperature. However, it is dependent on the DOC ξ. This is taken into account by using an equation following [22]:

(5) $$\log\left(G^{\infty }\left(\xi \right)\right)=C_{g}+\frac{D_{g}}{\left(1+\exp\left(\left(\xi_{gel}-\xi \right)/F_{g}\right)\right)},$$

with the model parameters $C_{g}$, $D_{g}$, and $F_{g}$.

#### 2.3.4. Disequilibrium Behaviour—Time-Dependent

To model the viscoelastic behaviour of the investigated EP, Maxwell-elements, each consisting of a spring with stiffness *G* and a dashpot with viscosity η, are used to derive the governing equations. This allows for the decomposition of the total strain γ in the purely elastic part $\gamma^{e}$ and the purely viscous part $\gamma^{v}$ per

(6) $$\gamma =\gamma^{e}+\gamma^{v}.$$

The material’s stress response $\tau^{ov}$ to a deformation is calculated according to

(7) $$\begin{array}{cc} \tau^{ov} & =G\;\gamma^{e}\overset{\left(6\right)}{=}G\;\left[\gamma -\gamma^{v}\right]\;\mathrm{or} \end{array}$$

(8) $$\begin{array}{cc} \tau^{ov} & =\eta \;{\dot{\gamma }}^{v}. \end{array}$$

Since this stress would relax to 0 Pa if there is no change in the strain state and if a sufficiently long time passes, it is referred to as overstress, describing its deviation from the equilibrium state. It is therefore denoted with the superscript ov.

Differentiating (7) with respect to time yields

(9) $$\frac{d(7)}{dt}\Rightarrow {\dot{\tau }}^{ov}=\underset{I}{\underset{︸}{G\;[\dot{\gamma }-{\dot{\gamma }}^{v}]}}+\underset{\mathrm{II}}{\underset{︸}{\frac{\partial G}{\partial \xi }\frac{d\xi }{dt}\;\left[\gamma -\gamma^{v}\right]}},$$

taking into account the indirect dependency of the spring’s stiffness on time via a change in the DOC in summand II.

From (9), it becomes clear that this might lead to changes in the stress state ${\dot{\tau }}^{ov}$ without changes of the external deformation $\dot{\gamma }$ or the internal subdivision of the strain into elastic and viscous parts $\dot{\gamma^{v}}$. Solely the process of curing $\frac{d\xi }{dt}$ and its effect on the fundamental material’s property *G* induce this change.

Reordering of (8) and substituting (7) leads to

(10) $${\dot{\gamma }}^{v}=\underset{I}{\underset{︸}{\frac{\tau^{ov}}{\eta }}}=\underset{\mathrm{II}}{\underset{︸}{\frac{G}{\eta }\left(\gamma -\gamma^{v}\right)}}.$$

Solving (7) for the viscous strain $\gamma^{v}$ results in:

(11) $$\gamma^{v}=\gamma -\frac{\tau^{ov}}{G}.$$

Inserting (10) and (11) into (9) allows for the complete elimination of $\gamma^{v}$ and the expression of ${\dot{\tau }}^{ov}$ is solely dependent on $\tau^{ov}$, $\dot{\gamma }$, and the material properties G,η:

(12) $${\dot{\tau }}^{ov}=\underset{I}{\underset{︸}{G\;\dot{\gamma }}}-\underset{\mathrm{II}}{\underset{︸}{\left(\underset{\mathrm{II}.i}{\underset{︸}{\frac{G}{\eta }}}-\underset{\mathrm{II}.\mathrm{ii}}{\underset{︸}{\frac{\partial G}{\partial \xi }\frac{d\xi }{dt}\frac{1}{G}}}\right)\tau^{ov}}}\;.$$

In (12), the immediate change of stress due to deformation is captured in (12)-I. Expression (12)-II describes the relaxation process driven by the current stress $\tau^{ov}$. Expression (12)-II.i shows the relaxation effect due to strain redistribution between spring and dashpot, whereas (12)-II.ii captures the changes in stiffness due to progressive curing.

#### 2.3.5. Final Model Assembly

**Figure 1:**
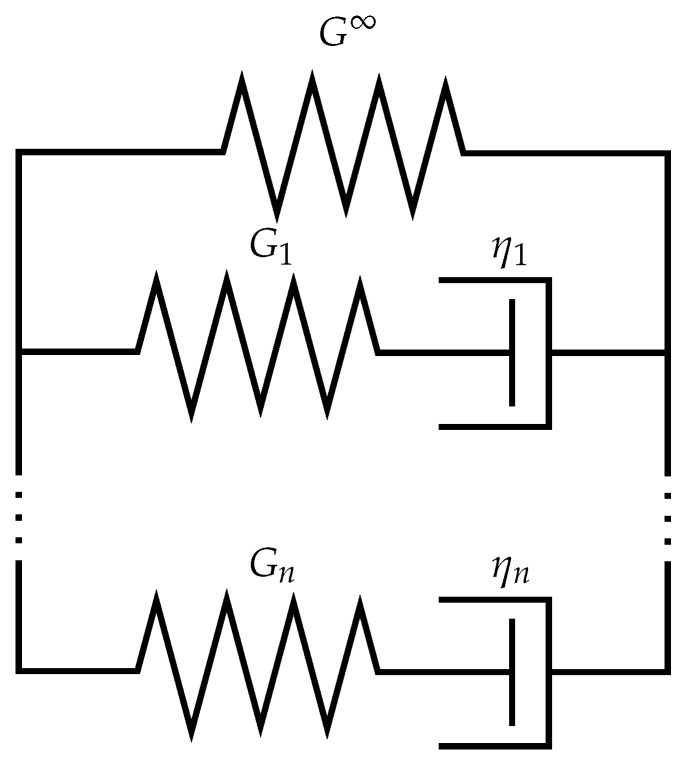
Schematic of rheological substitute for entire material behaviour.

To consider all previously identified effects and model the strong non-linearity observable in the experiment, the rheological substitute in Figure 1, showing a generalised Maxwell-material, is taken as basis for the final model.

The single spring represents the time-independent long-term behaviour with the equilibrium stress $\tau^{eq}$. The parallel combination of *n* Maxwell elements captures the time dependence in the overstresses $\tau_{i}^{ov}$ in the *i*th element, allowing for more realistic behaviour by the arbitrary number of elements. To incorporate temperature effects, the time–temperature analogy in form of the shift factor $a_{T}$ is applied to the Maxwell elements. Given the parallel connection of the elements,

(13) $$\begin{array}{cc} \gamma & =\gamma_{0}=\gamma_{i}=\gamma_{n}\;\mathrm{and} \end{array}$$

(14) $$\begin{array}{cc} \tau & =\tau^{eq}+\sum_{i=1}^{n}\tau_{i}^{ov} \end{array}$$

hold.

In extension of (4) and (12), this leads to the final set of differential equations (DEs) describing the material’s behaviour:

(15) $$\begin{array}{cc} {\dot{\tau }}^{eq} & =G^{\infty }\;\dot{\gamma }\\ {\dot{\tau }}_{i}^{ov} & =G_{i}\;\dot{\gamma }-\left(a_{T}\left(T,\xi \right)\frac{G_{i}}{\eta_{i}}-\frac{\partial G_{i}}{\partial \xi }\frac{d\xi }{dt}\frac{1}{G_{i}}\right)\tau_{i}^{ov};\;i=1\ldots n, \end{array}$$

where the term ηiGi is often replaced by the resulting relaxation time $\tau_{i}$.

## 3. Results

The following section is structured in accordance with the suggested framework which is composed of (i) testing, (ii) modelling, (iii) calibration, and (iv) validation. Based on the experimental results, the required model parameters are identified (see Section 3.1). Furthermore, a thorough model analysis is performed, which includes both a calibration (see Section 3.2.1) and a validation of the suggested approach (see Section 3.2.2).

### 3.1. Parameter Identification

The parameter identification to fit models to the experimental data has been carried out with the gradient-based nonlinear least-square optimisation algorithm scipy.optimize.least_squares implemented in [31].

#### 3.1.1. Determination of Fully Relaxed Modulus

To determine the model parameters for the equilibrium stiffness $G^{\infty }$, the measured time-dependent storage moduli from the DMA experiments described in Section 2.2.1 are transferred to a DOC-dependent scale. Since it is assumed that stresses built up prior to gelation can relax completely, such storage moduli are neglected and the results are solely evaluated for ξ>0.68. Furthermore, the results are clipped at a storage modulus threshold of 4 MPa. Measurements beyond this threshold are dominated by compliance effects of the device, leading to erroneous results (cf. Section 2.2.1, [21,24]).

As described in Section 2.3.3, the equilibrium modulus is modelled independent of temperature as a consequence of the assumption of thermorheological simple behaviour. Therefore, the results obtained at 80 and 100 °C are combined into one single dataset. This dataset is extended further by adding the *G*-value obtained at the end of the relaxation experiment on the fully cured material at the highest tested temperature of 125 °C.

**Figure 2:**
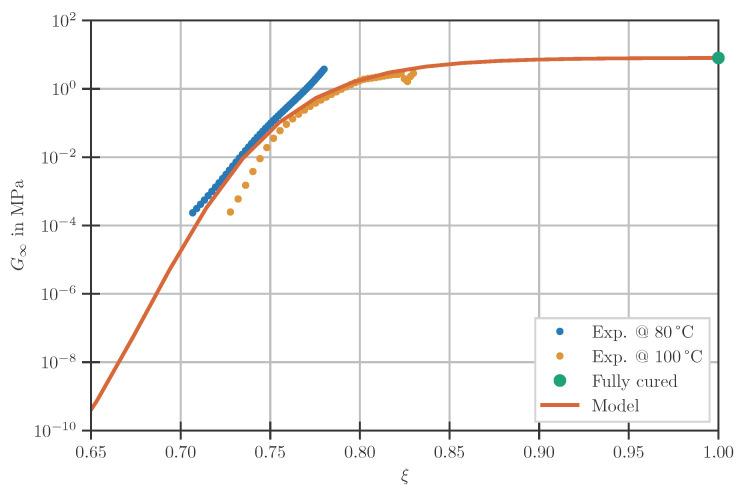
Fully relaxed modulus with the used model.

Since the sub-datasets consist of a very different number of points but should all have the same impact on the final fit, a weighted fit is conducted. The weights are identical throughout each sub-dataset and defined as the inverse of the number of datapoints in the respective subset. The fitting is carried out on the logarithm of the residuals, to ensure that deviations at low absolute values are not overshadowed by those at high absolute values. This leads to the model parameters given in Table 3. The comparison of experimentally determined datapoints and the fitted model is depicted in Figure 2.

#### 3.1.2. Analysis of Relaxation Experiments

**Figure 3:**
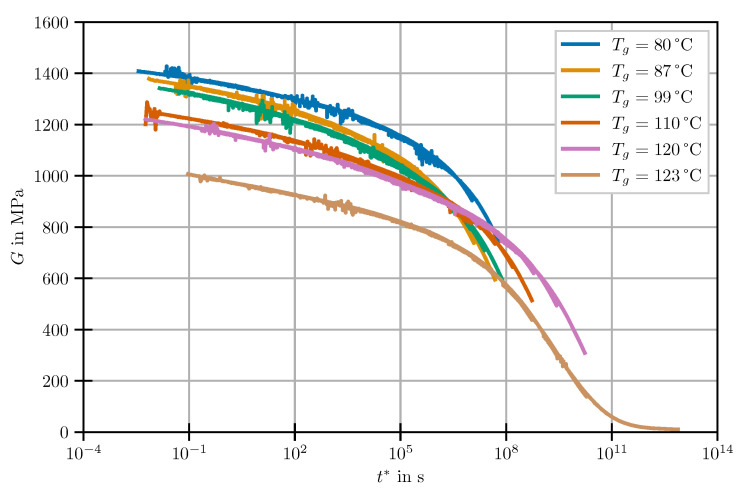
Master curves for different states of partial cure at reference temperature 40 °C.

Given the assumption of a thermorheologically simple material, the time-dependent shear moduli determined in the relaxation experiments were assembled to individual master curves having a uniform reference temperature of 40 °C. For this purpose, shift factors according to (2) were determined for all temperatures and applied to the respective experimental times. This leads to one single equivalent relaxation master curve per DOC level with drastically increased holding time. For the sake of clarity, it should be pointed out that the cure state of the resin will be indicated by its $T_{g}$ determined in DSC scans and described by the DiBenedetto equation [13]. The $T_{g}$ is chosen since the measured residual enthalpy which is usually used to calculate the DOC yields high scatter, especially at high DOC-levels. The results of the master curves are shown in Figure 3.

The stress relaxation is shifted towards longer times at increasing $T_{g}$. Furthermore, the shear relaxation modulus in the glassy regime decreases with increasing DOC. Within the regarded range of $T_{g}$ between 80 and 123 °C, the stiffness is reduced by almost 40%. This significant decrease is in line with previously published findings on cure-dependent properties of EP. According to the structure–property-relationships discussed in [32,33,34], the reduction of $G^{0}$ with cure is caused by the reduced chain mobility of the crosslinked molecular structure. This leads to a reduced packing density and a higher amount of free volume during cool-down into the glassy state. However, authors that focus on material modelling rarely reported a negative correlation between DOC and $G^{0}$. It was either not clearly found during the measurements [17,18,22,23], discarded [20] or attributed to measurement uncertainties [25]. In this context, the device compliance which limits the measurable stiffness of parallel-plate geometries [12,21] may play an important role. Due to the pronounced cure-dependence of $G^{0}$, it must be concluded that the often-adopted approach of time–cure analogy [17,18,22,23] is not applicable to the selected resin system.

**Figure 4:**
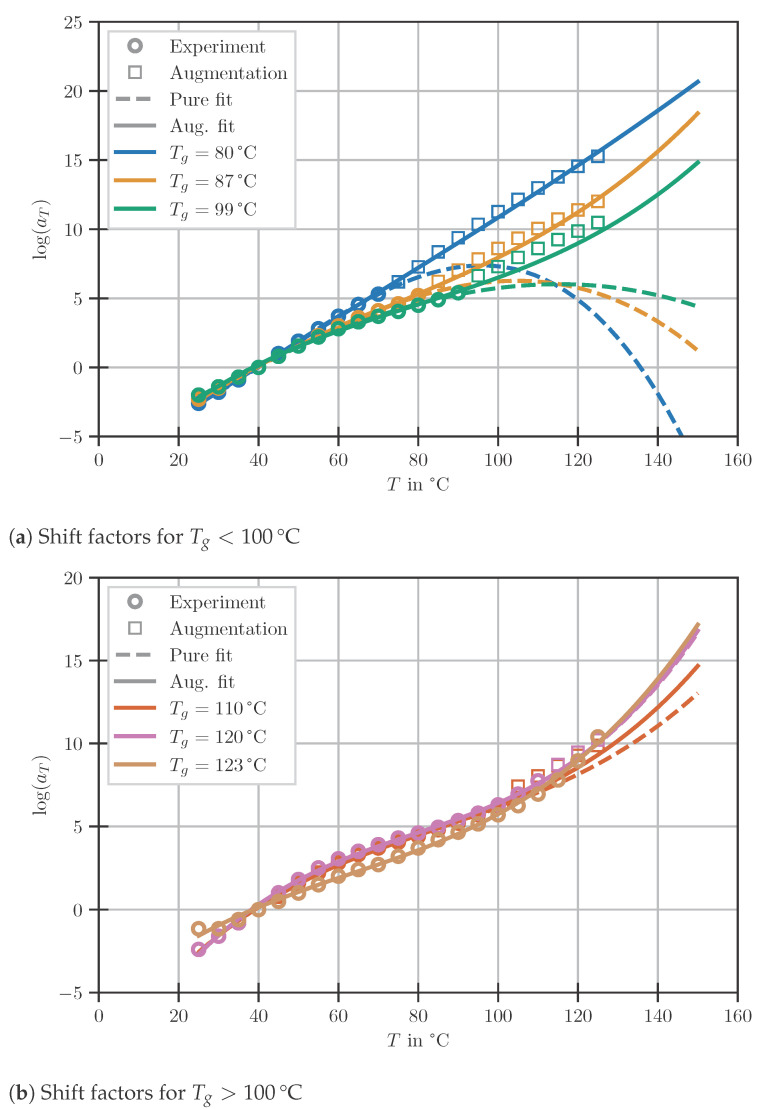
Shift factors vs. temperature for the tested DOC levels.

To generalise and gain the ability of predicting shift factors for arbitrary temperatures, the cubic model defined in (3) is used. As shown in Figure 4, the model fit by a standard least square approach is in excellent agreement with the existing data. However, from Figure 4a, it becomes clear that the model is not suitable for extrapolation since no experimental data are available for low $T_{g}$ at high experimental temperature since experimental temperatures must be limited to avoid post-cure [35]. Rather than capturing the trend of a steep monotonic increase of $a_{T}$ with temperature, a flattening or even decline of the curve is predicted.

This effect is much less pronounced at higher DOC levels, as shown in Figure 4b, since more data points are available, reducing the necessity of extrapolating extensively to high temperatures. To prevent such erroneous predictions, the data are augmented using a standard Williams–Landel–Ferry (WLF) fit, guaranteed to rise with temperature, to generate points at high temperatures. In the subsequent least square fit, deviations from the artificial data are weighted by a factor of 0.1. However, this slight adjustment to the fitting procedure leads to a much better generalisability of the model whilst maintaining the excellent interpolation capabilities. Temperatures outside the range of 25 and 150 °C are not taken into account for this model as they are of negligible importance for the manufacturing process. The determined parameters are given in Table 4.

**Figure 5:**
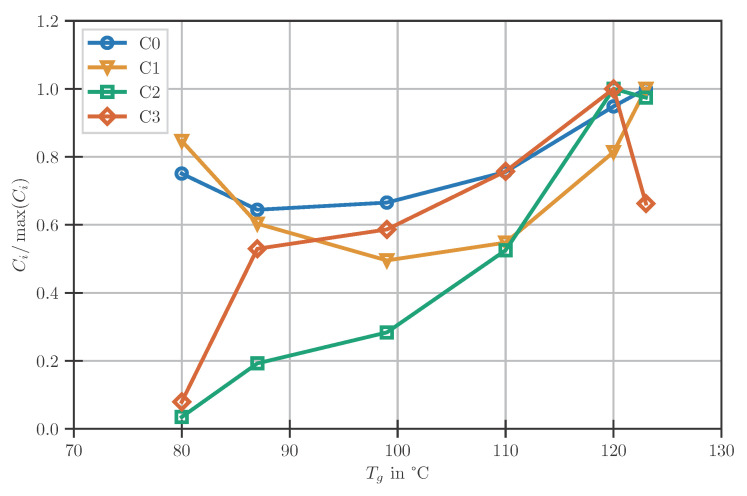
Change of the parameters of the temperature models with DOC.

The dependency of the resulting fitting parameters $C_{i}$ on the current $T_{g}$ is shown normalised to the respective maximum in Figure 5. As can be seen, no clear trend of the parameters with respect to the DOC is present. Especially the inconsistent changes in the slopes of the connecting straight lines complicate the derivation of a generalising model. Therefore, the dependency on the $T_{g}$ is taken into account by keeping the determined points and interpolating linearly between them.

The relaxation experiments with partially cured specimen were conducted solely at temperatures at which no curing occurs. This was ensured by keeping the experimental temperature at least 10 K below the respective specimen’s $T_{g}$ [35]. Therefore, in (12), the factor dξdt and, as a consequence, the summand II.ii are zero throughout all relaxation experiments. This leads to a simplified set of DEs which allows for an analytical description of the momentary material’s stiffness G(t) in the form of a Prony-series:

(16) $$\begin{array}{cc} G(t) & =G^{\infty }+\sum_{i=1}^{n}G_{i}\exp\left(\frac{G_{i}t}{\eta_{i}}\right)\\ & =G^{\infty }+\sum_{i=1}^{n}G_{i}\exp\left(\frac{t}{\tau_{i}}\right). \end{array}$$

For modelling purposes, it is often beneficial to normalise the governing coefficients as it allows for the separation of absolute values and principal shape of the master curve. Therefore, normalised parameters are defined per gi=GiG0. This and expressing the absolute value in terms of $G^{0}$ instead of $G^{\infty }$, with

(17) $$G^{0}=G^{\infty }+\sum_{i=1}^{n}G_{i},$$

leads to

(18) $$G\left(t\right)=G^{0}\left(1-\sum_{i=1}^{n}g_{i}\;\cdot \;\left[1-\exp\left(\frac{t}{\tau_{i}}\right)\right]\right),$$

which is used for further parameter identification.

When dealing with a fast-curing EP, it is unfeasible to obtain reliable experimental data at low DOC and elevated temperatures, since post cure sets in before measurements can be performed. This results in incomplete master curves (cf. Figure 3) missing information on the long-term behaviour. This leads to Prony-series fitted on the pristine experimental data significantly overestimating $G^{\infty }$, since no information on a further decrease of stiffness is present in the data.

**Figure 6:**
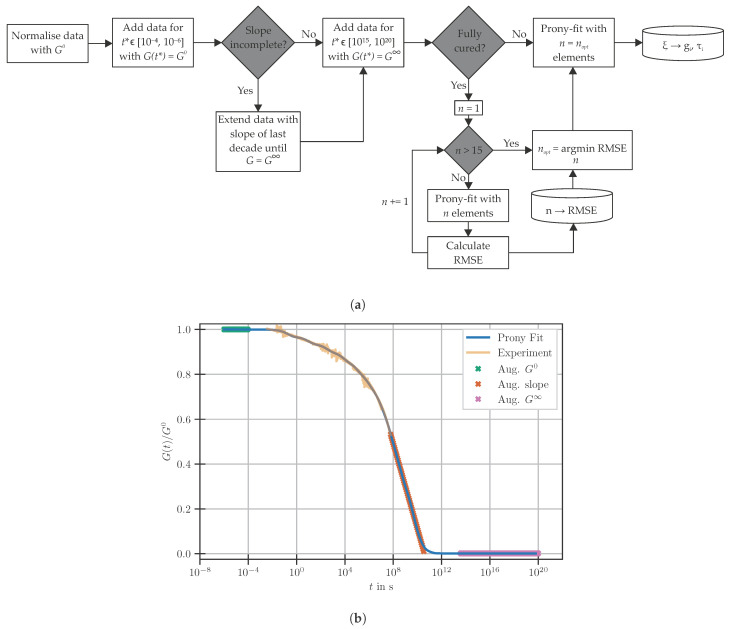
Fitting of the master curves at partial cure. (**a**) Workflow to augment partial datasets and determine Prony-parameters. (**b**) Added Data for Prony-series fitting, experimental data at $T_{g}=80\;{}^{{}^{\circ}}C$.

To overcome this hindrance and to meet the normalised representation of the Prony-parameters, partial experimental datasets are augmented. In order to obtain master curves that can be reliably fitted by a Prony-series, the workflow shown Figure 6a is employed. The added data points are spaced equally on the time axis in the logarithmic domain. An example of the data used for fitting the Prony-series at $T_{g}=80\;{}^{{}^{\circ}}C$, the lowest $T_{g}$ investigated within this work, is shown in Figure 6b.

### 3.2. Model Analysis

To ascertain the model’s capabilities, two methods for solving the DEs are investigated and the respective results compared to the experimental ones. Firstly, the direct numerical solution with initial conditions using an implicit time integration scheme and the algorithm scipy.integrate.solve_ivp, implemented in [31], is employed. This method is well-suited for geometrically simple use cases in which the strain loading is known beforehand. In such cases, the direct solution can be set up easily and yields accurate results quickly. Secondly, a spatial discretisation with boundary conditions (BCs) and a subsequent numerical solution in the discretised region using Abaqus/Standard [36] with an external UMAT containing the DEs is used. This method is suitable for arbitrarily complex cases; however, model setup and evaluation are more complex and time-consuming. In the former case, the DEs are investigated with and without consideration of the immediate effects of curing induced changes in stiffness, i.e., (12)-II.ii. In the latter case, these effects are not taken into account since implementation and solution would both be drastically complicated and impeded.

#### 3.2.1. Calibration

**Figure 7:**
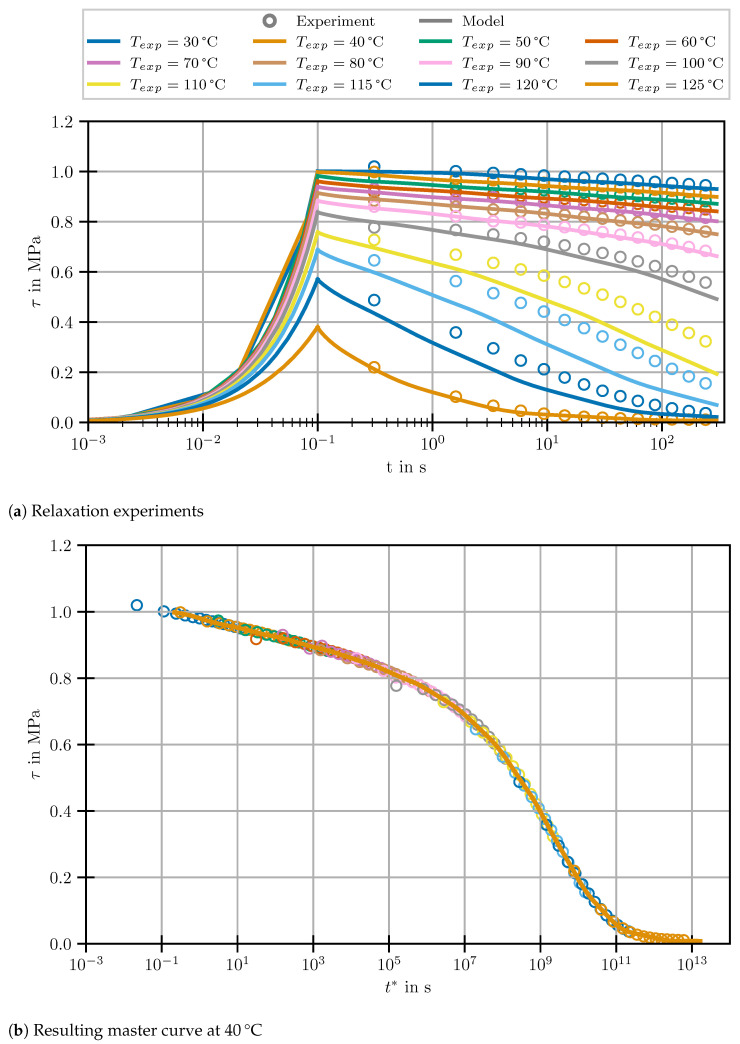
Comparison of experiment and model predictions for relaxation and master curve at 40 ${}^{{}^{\circ}}C$ of the fully cured specimen.

To verify that the material model is suitable for modelling the investigated EP and all parameters have been identified satisfactorily, the relaxation experiments used for assembling the master curve are numerically recreated. Furthermore, a relaxation experiment investigating the entire master curve at the reference temperature of 40 °C over more than 14 decades of time is simulated. The results for the fully cured material are shown in Figure 7a for the individual relaxation experiments and in Figure 7b for the master curve. The same verification procedure has been applied to all investigated states of partial cure. The respective results are presented in Appendix A. From the results for different DOCs, it becomes clear that the cure-dependence of $G^{0}$ is captured well by the model.

As can be seen, the model captures the master curve, which was used to fit the model parameters excellently throughout the entire time range. In case of the relaxation experiments, very good agreement for low to medium temperatures can be observed. From $T_{exp}$ = 100 to 120 °C, however, model and experiment deviate slightly. At the highest temperature of 125 °C, the agreement between model and experiment is excellent again. Given that the deviations only occur in the relaxation experiment, but do not persist in the master curve, they can be attributed to imperfections in the fit of the shift factors $a_{T}$.

Throughout all relaxation experiments, the effect of cure-induced stiffness changes (Equation (12)-II.ii) is negligible compared to relaxation effects (Equation (12)-II.i). This is a result of the minimal $T_{g}$ tested being 80 °C. According to the findings in [35], it leads to a minimum temperature for curing of $T_{cure,min}=70\;{}^{{}^{\circ}}C$. The corresponding minimum shift factor is $a_{T,cure,min}=265,339$. Therefore, the equivalent time $t^{*}$ for relaxation is so long that slight changes in the stiffness do not noticeably contribute to the material’s stress response under the investigated loading conditions.

#### 3.2.2. Validation

**Figure 8:**
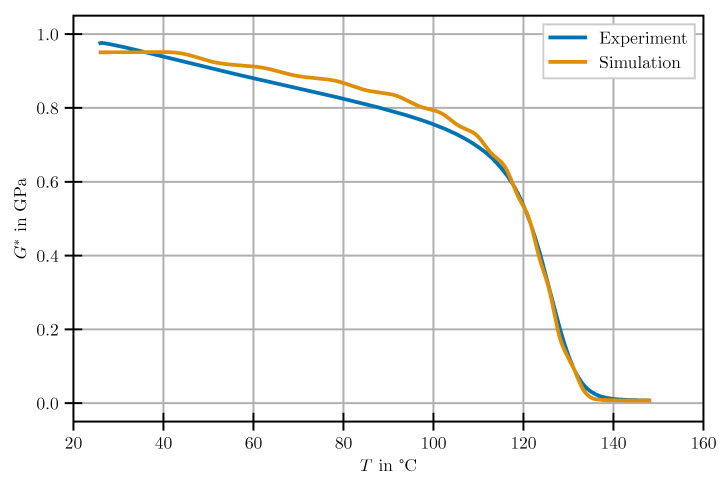
Investigation of the predictive capabilities of the developed model for the novel type of experiment—DMA.

In order to investigate the predictive capabilities of the developed model, the insights obtained by relaxation experiments are applied to the numerical reproduction of the DMA experiment described in Section 2.2.3. It is analysed using the UMAT implementation in Abaqus/Standard. The simulation is set up with an ideal state of pure sinusoidal shear deformation with an amplitude of 0.1% and frequency of 1 Hz as BC. The specimen’s temperature is defined to rise from 25 to 150 °C with a heating rate of 2 Kmin. The predicted complex moduli are shown together with their experimental equivalents in Figure 8.

Comparison of experimental and simulation results for the fully cured material shows excellent agreement throughout the entire investigated temperature range.

## 4. Discussion

Process-induced residual stresses in PMC determine a range of composite key properties such as the part shape as well as fibre–matrix-adhesion, fracture, and fatigue behaviour. The numerical prediction of the resulting stress state requires the development and implementation of a constitutive model for the cure-dependent viscoelastic properties of the matrix. Especially if fast-curing matrix resins are addressed, this involves challenging experiments and a material-specific modelling approach taking into account the special resin characteristics. In this work, a comprehensive framework including (i) testing, (ii) modelling, (iii) calibration, and (iv) validation is suggested which pays special attention to the determination of high-fidelity experimental data, their thorough discussion taking into account structure–property-relationships as well as model validation using an independent dataset.

The material model captures the dependency of the selected fast-curing EP on DOC, temperature and time. The development of the relaxed modulus $G^{\infty }$ was measured at different isothermal temperatures in parallel-plate rheology. It was found that the relaxed modulus $G^{\infty }$ increases with DOC, which is in agreement with previous observations. Hence, it is represented by an established model from the literature. Additional isothermal experiments were performed in relaxation mode using solid rectangular rods having a predefined partial DOC. The time- and temperature-dependent datasets were assembled to master curves, one per investigated DOC. The assumption of a thermorheologically simple material and the following time–temperature-analogy were employed. Representing shift factors were modelled using a cubic approach. Its general form allows to accurately capture the observed change in the fundamental dependency of shift factors on the temperature. In order to achieve models with physically sound extrapolation capabilities, partial experimental datasets corresponding to low DOCs were augmented using the established WLF approach.

It was found that the time–cure analogy does not hold true for the selected resin system as the instantaneous modulus $G^{0}$ shows a pronounced negative correlation with cure. This behaviour can be attributed to the reduced chain mobility of the crosslinked molecular structure in the glassy state. Hence, the specific relaxation behaviour at different investigated states of partial curing was modelled using individual Prony-series. To obtain physically sound results even in the case of incomplete data, an augmentation workflow was developed and employed. This led to outstanding results regarding the reproduction and extension quality of the model for the conducted relaxation experiments.

Furthermore, the model has been shown to be in good agreement with the experiments conducted for parameter identification under varying process-like conditions. The fully relaxed modulus is represented well in the entire DOC-range corresponding to a solid state. Furthermore, the behaviour of the shift factors for time–temperature superposition with regard to changes in temperature and DOC is captured with high accuracy. The combination of these two aspects in combination with DOC-dependent Prony-series leads to an excellent agreement between relaxation experiments in a temperature range of 25 to 125 °C as well as the derived master curve at 40 °C and the corresponding model predictions. In the case of the relaxation experiments, the model even allows for an analysis of the load application phase. Hence, it has been verified that the model is capable of capturing and reproducing all discussed dependencies controlled and investigated throughout the manufacturing process with high accuracy.

To analyse the model’s predictive capabilities, the following points were investigated in a DMA on a fully cured specimen:

- Permanent changes of strain and strain rate even including sign changes in both quantities;
- Change of the temperature with a constant rate throughout the experiment;
- A very long period of time to be investigated coherently.

All three aspects were found to be excellently captured by the model. The observable waviness of the simulated curves is a result of the discretisation of the material’s relaxation spectrum in Prony-elements.

This newly developed threefold combination of

1. high-fidelity, process-near testing,
2. tailored material modelling and calibration with
3. independent validation experiments

represents a consistent framework for the in-depth analysis of thermoset matrix systems for PMCs. Future works should focus on verifying the transferability to different matrix systems and on uniting the currently individual models into one unified theory.

## Figures and Tables

**Table 1:** Overview of post-cure schedules for preparation of SRF specimens initially cured at 60 ${}^{{}^{\circ}}C$ and resulting $T_{g}$ determined by subsequent DSC scans.

Post-Cure Temperature in ${}^{{}^{\circ}}C$	Post-Cure Dwell-Time in min	Resulting $T_{g}$ in ${}^{{}^{\circ}}C$
-	-	80.5
75	10	87.1
85	10	99.6
95	10	109.7
105	10	120.8
130	10	123

**Table 2:** Parameters of the model for the current reaction rate.

*m*	$n_{1}$	$A_{1}$ in log(1s)	$E_{1}$ in kJmol	$n_{2}$	$A_{2}$ in log(1s)	$E_{2}$ in kJmol
1.47	1.41	1.43	26.18	0.62	6.99	67.65
$C_{1,\mathrm{diff}}$	$C_{2,\mathrm{diff}}$ **in K**		$k_{1,\mathrm{diff}}^{*}$ **in log(1s)**		$k_{2,\mathrm{diff}}^{*}$ **in log(1s)**	
12	50		−3		−5.5	

**Table 3:** Parameters of the model for the equilibrium modulus.

$C_{g}$ in log(MPa)	$D_{g}$ in log(MPa)	$F_{g}$
−14.160	15.067	0.0386

**Table 4:** Parameters of the cubic shift model for the tested $T_{g}$.

	$T_{g}$ in °C					
	80	87	99	110	120	123
$C_{0}$	7.2	6.2	6.4	7.3	9.1	9.6
$C_{1}$	0.18	0.13	0.11	0.12	0.17	0.21
$C_{2}$	7.8 × 10^−5^	4.3 × 10^−4^	6.4 × 10^−4^	1.2 × 10^−3^	2.2 × 10^−3^	2.2 × 10^−3^
$C_{3}$	1.4 × 10^−6^	9.7 × 10^−6^	1.1 × 10^−5^	1.4 × 10^−5^	1.8 × 10^−5^	1.2 × 10^−5^

**Figure A1:**
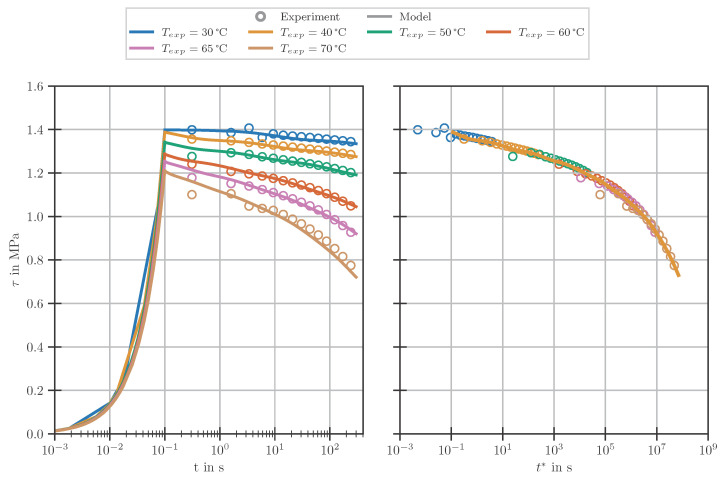
Comparison of relaxation experiments/master curve and model for $T_{g}=80\;{}^{{}^{\circ}}C$.

**Figure A2:**
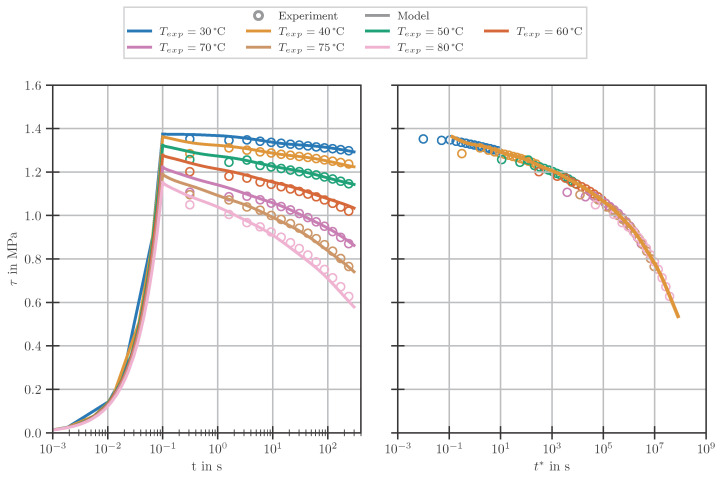
Comparison of relaxation experiments/master curve and model for $T_{g}=87\;{}^{{}^{\circ}}C$.

**Figure A3:**
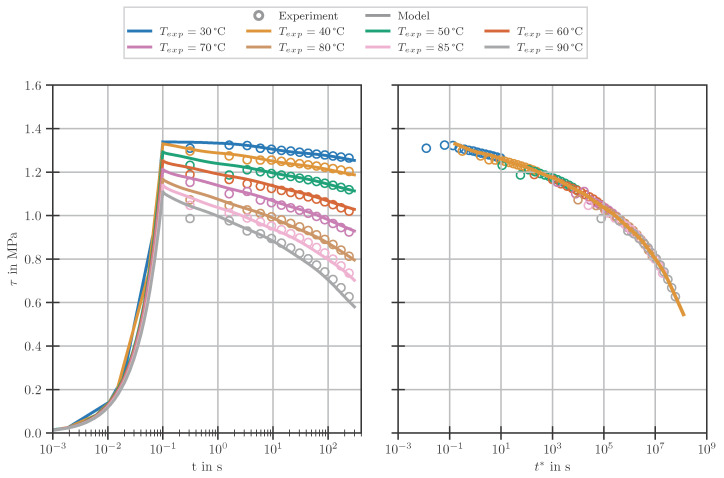
Comparison of relaxation experiments/master curve and model for $T_{g}=99\;{}^{{}^{\circ}}C$.

**Figure A4:**
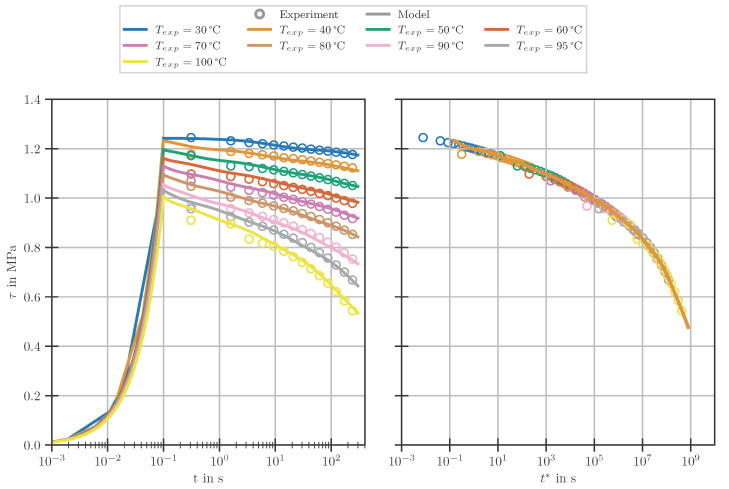
Comparison of relaxation experiments/master curve and model for $T_{g}=110\;{}^{{}^{\circ}}C$.

**Figure A5:**
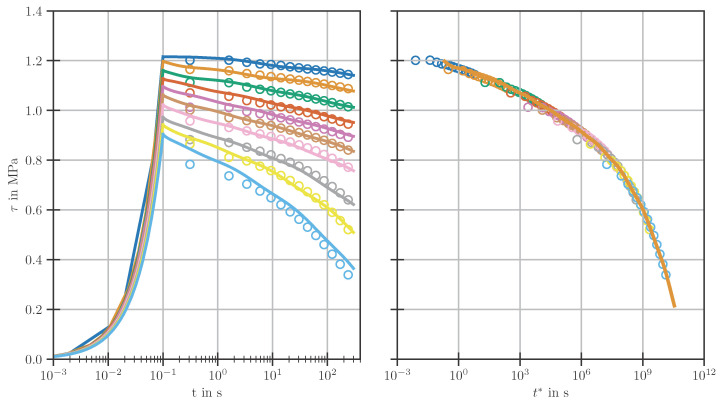
Comparison of relaxation experiments/master curve and model for $T_{g}=120\;{}^{{}^{\circ}}C$.

**Figure A6:**
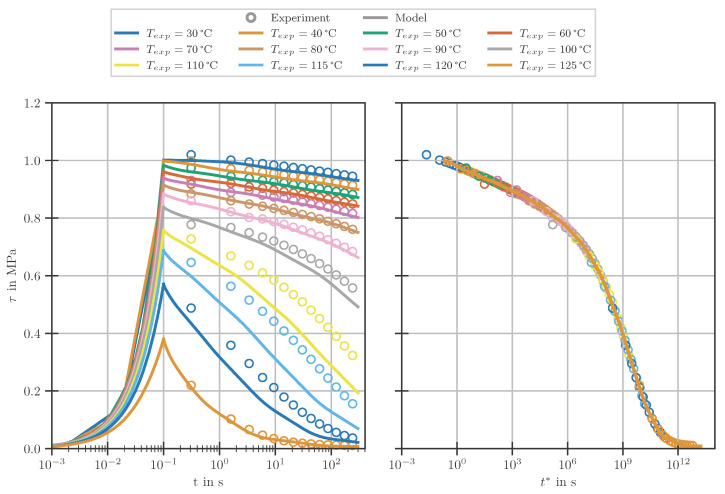
Comparison of relaxation experiments/master curve and model for $T_{g}=123\;{}^{{}^{\circ}}C$.

## Data Availability

The data presented in this study are available on request from the corresponding author.

## Abbreviations

The following abbreviations are used in this manuscript:

**BC**	boundary condition
**DE**	differential equation
**DMA**	dynamic mechanical analysis
**DOC**	degree of cure
**DSC**	differential scanning calorimetry
**EP**	epoxy resin
**PMC**	polymer matrix composites
**RTM**	resin transfer moulding
**SRF**	solid rectangular fixture
$T_{g}$	glass transition temperature
**WLF**	Williams–Landel–Ferry
